# Consultative engagement of stakeholders toward a roadmap for African language technologies

**DOI:** 10.1016/j.patter.2023.100820

**Published:** 2023-08-11

**Authors:** Kathleen Siminyu, Jade Abbott, Kọ́lá Túbọ̀sún, Aremu Anuoluwapo, Blessing K. Sibanda, Kofi Yeboah, David Adelani, Masabata Mokgesi-Selinga, Frederick R. Apina, Angela Thandizwe Mthembu, Arshath Ramkilowan, Babatunde Oladimeji

**Affiliations:** 1 Masakhane Research Foundation, Jamii Town, Kilifi 80108, Kilifi County, Kenya; 2 Mozilla Foundation, 331 East Evelyn Avenue, Mountain View, CA 94041, USA; 3 Lelapa.AI, 5 Lower Road, Johannesburg 2157, Gauteng, South Africa; 4 Yoruba Names, Lekki-Epe Express Road, Lagos 101245, Nigeria; 5 UCL Centre for Artificial Intelligence, 90 High Holborn, London WC1V 6BH, UK; 6 Parliament of the Republic of South Africa, 120 Plein Street, Cape Town 8000, Western Cape, South Africa; 7 Belltro, 10th Road Mwanga Street, Dodoma, Tanzania; 8 A24 Media, Kempton Park, Johannesburg, Gauteng, South Africa; 9 Helm, 50 Smits Road, Randburg 2196, Gauteng, South Africa; 10 Amazethu, Stockton CA 95219, USA

**Keywords:** Africa, natural language processing, machine learning, technology, language technology, African langauges, participatory research

## Abstract

There has been a rise in natural language processing (NLP) communities across the African continent (Masakhane, AfricaNLP workshops). With this momentum noted, and given the existing power asymmetries that plague the African continent, there is an urgent need to ensure that these technologies move toward shared goals between organizations and stakeholders, not only to improve the representation of African languages in cutting-edge NLP research but also to ensure that NLP research enables technological advances toward human dignity, well-being, and equity for those who speak African languages. This study investigates the motivations, focus, and challenges faced by various stakeholders who are at the core of the NLP process. We perform structured stakeholder identification to identify core stakeholders in the NLP process. Interviews with representatives of these stakeholder groups are performed and are collated into relevant themes. Finally, a set of recommendations are proposed for use by policy and artificial intelligence (AI) researchers.

## Highlights

• This study describes stakeholders in the African natural language processing (NLP) space
• For each stakeholder a persona is compiled through qualitative interviews
• We extract important themes that are identified across personas
• Recommendations for stakeholders working in the African language ecosystem are provided

## The bigger picture

This study investigates the motivations, focus, and challenges faced by stakeholders at the core of the NLP ecosystem in an African context. We perform structured stakeholder identification to identify core stakeholders in the NLP process. Interviews with representatives of these stakeholder groups are performed, and output is collated into relevant themes. Personas are created and storytelling is employed to bring to life the experiences and perspectives of stakeholders while maintaining anonymity.

Finally, recommendations for the betterment of the African NLP ecosystem are proposed; these range from recommendations aimed at greater language content development to others aimed at priority areas for language technology development. The recommendations are for use by policy makers, AI researchers, and other relevant stakeholders.

## Graphical abstract

**Figure:**
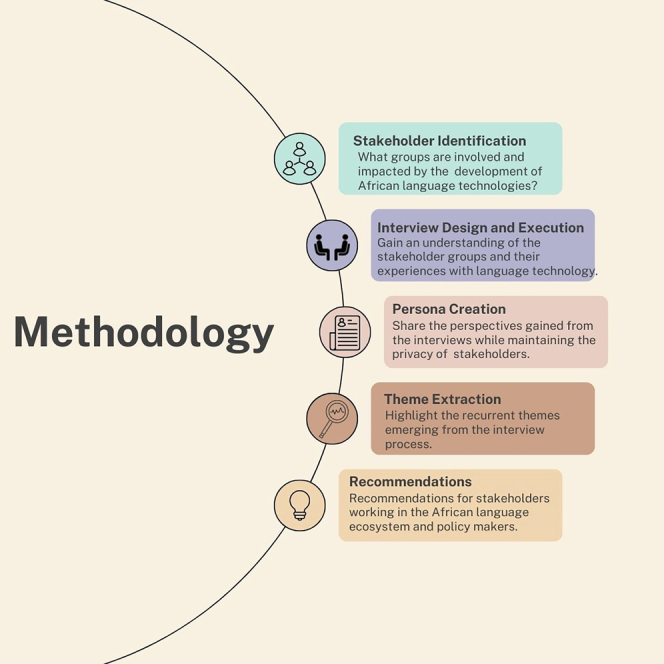

## Teaser

This study investigates the motivations, focus and challenges faced by stakeholders at the core of the NLP ecosystem in an African context. We perform structured stakeholder identification to identify core stakeholders in the NLP process. Interviews with representatives of these stakeholder groups are performed and collated into relevant themes. Finally, recommendations are proposed for use by policy makers, AI researchers, and other relevant stakeholders in aid of the betterment of the development of language content and language technology.

## Introduction

There has been a rise in machine learning and, more specifically, natural language processing (NLP) communities across the African continent, most notably, the success of the Masakhane community^1^ and two AfricaNLP workshops.^2^^,^^3^

While improving the representation of African languages in cutting-edge NLP research, it is vital that African NLP activities lead to greater access and better quality of life for populations that speak African languages.

The purpose of this study is to more deeply understand how different segments of the population might be affected by emerging NLP technologies in Africa. The resulting themes will be collected and analyzed, which can be used by policy and artificial intelligence (AI) researchers.

In the experimental procedures, we discuss our methodology, while we detail the identified stakeholders in the results and discussion. In the results and discussion, we also discuss the personas that are compiled from the interviews and we identify prevailing themes. We further list a set of recommendation and highlight the limitations of our study.

## Results and discussion

### Methodology

Our study consists of stakeholder identification followed by interviews with experiential experts. These expert interviews were compiled into personas, and the high-level themes were extracted and analyzed.

#### Stakeholder identification

Inspired by the Diverse Voices methodology,^4^ we asked the following questions to build up a list of relevant stakeholder groups to the AfricaNLP ecosystem:

• Which groups are likely to develop African NLP technologies?
• Which groups are likely to use African NLP technologies?
• Which groups are not likely to use African NLP technologies due to factors such as structural inequality in society, disinterest, or self-described technophobia?
• Which groups will be affected as African NLP technology becomes more pervasive, possibly decades into the future?
• Which groups are likely to be overlooked, based on the groups represented by the authors?

The stakeholder group list was circulated for comment within the Masakhane community at the weekly meeting and via the Slack to incorporate the wider Masakhane community’s contributions to the list. Masakhane is an open community of researchers that spurs research in NLP for African languages, which boasts research contributors from across the African continent from a wide diversity of backgrounds.^1^

Despite performing full stakeholder identification, the density of valuable insights we have retrieved from these interviews means we report only on the insights from the groups likely to develop African NLP technologies. Future work will report on insights from other stakeholder groups.

#### Interviews

To gain understanding of the stakeholder groups, we conducted interviews with two or three experiential experts for each group. Some interviewees fit into multiple stakeholder groups and so were asked questions for those stakeholder groups. In total, 12 experts were interviewed. Interviews took place in English and were performed by the study facilitators, J.A. and K.S.

As per Magassa et al.,^4^ we defined experiential experts as people speaking from personal lived experience, from institutions supporting the stakeholder group, or as social support to persons of lived experience.

The interview questions aimed to understand each stakeholder group in the following broad categories: (1) understand their perception of African NLP technology, (2) understand their role and the largest challenges associated with that role, and (3) learn the role of language technology in their work processes and explore how greater African language innovation might support their work. The questions were shared with the Masakhane community for feedback and contribution. The full list of questions is included in the supplemental experimental procedures. The interviews were conducted via Zoom, for 1 h, and were recorded with permission of the interviewees and transcribed. Where Zoom was not available, arrangements were made for the interviews to be performed via telephone or in person. The study facilitators offered authorship to all interviewees on papers published as a result of their interviews (in which this paper is included). The majority of the interviewees accepted co-authorship and were included in the editing of this paper. This is in line with the Masakhane Authorship Guidelines, which include editing and sharing lived experiences as a means of contributing to research.

To recruit the experiential experts for the interviews, the Masakhane community was asked if they could suggest experts for each of the stakeholder groups during a regular weekly meeting over three consecutive weeks as well as via the Masakhane Slack account. For sensitive stakeholder groups, institutions serving these stakeholders groups were approached. In addition, geographic, linguistic, and gender diversity of candidates informed the selection of experts.

### Stakeholders

The following section outlines the identified stakeholders as a result of the methodology defined under “stakeholder identification.” Unless explicitly stated, all of the stakeholder groups are of African origin.

#### Groups involved in development of African NLP technologies

This group includes individuals who contribute to the development of NLP tools, either directly by their actions or indirectly, as the output of their actions then feeds into the development of the technologies. Members of this group are often publicly perceived as the most at risk of being replaced by the technology as it becomes more sophisticated and human-like (although in practice this is rarely the case).

Nekoto et al.^1^ identify the agents involved in the creation of machine translation technologies. We abstract their agents to NLP more generally, as follows:

• Content creators consist of journalists, copywriters, creative writers, and technical writers who produce content in or about a language. Content creators are included in the NLP process, whether directly (when tasked with creating content for NLP projects directly) or indirectly (where their work might be scraped from the web or digitized).
• Language practitioners we define as the annotators, translators, and transcribers who may have formally studied languages and work to annotate, transcribe, or translate content, which includes translators, crowd-workers, or linguists. We have also included evaluators who measure and analyze performance of an NLP model before deployment, due to the overlap noted between individuals who evaluate and those who annotate, translate, and transcribe.
• Content curators select content for inclusion in a certain form of final output. They may be dataset curators who decide what gets included in a dataset, editors who decide what gets published in an online newspaper, or even data governance experts who decide what data can and should be accessed, and how.
• Language technologists use datasets and computational linguistic techniques to produce NLP models, including software engineers, NLP practitioners, and linguists.
• Language innovators are the entrepreneurs who are using language technologies to solve problems in various contexts. This agent is in addition to those identified in Nekoto et al.^1^

#### Groups likely to use African NLP technologies

In this group, we identified direct and indirect users of language technology. Direct users are stakeholders by whom language or communication is relied upon to perform their job, but is not the core focus on the job, such as, education, legal, public relations (PR), customer service, media, government, health, commerce, and market research. While indirect users do not necessarily engage with the technology itself, they instead engage with the artifacts generated by the technology, for example, the public, who might engage with machine-translated media in printed books or speech-to-text in subtitled films.

#### Groups unlikely to use African NLP technologies

To identify which stakeholders are not likely to use the technology of interest, we consider a number of accessibility dimensions and use the dimensions to help identify population groups:

• Income (device and connectivity costs)
• Internet access (function of location and income)
• Literacy and education access (for text-based NLP)
• Digital literacy
• Hearing ability (for speech-based NLP)
• Eyesight ability (for text-based NLP)
• Language (their language would need to be supported)

We use the above dimensions to identify marginalized and underrepresented groups that are often left out of the technology conversation in Africa, including the hearing or eyesight impaired; individuals below the poverty line; individuals relying on languages that do not have an established writing system or undocumented languages; those without connectivity to the internet; the illiterate; the elderly; children; women, especially rural women and single mothers; the unemployed; the incarcerated; people with disabilities; illegal immigrants; those living under oppressive governments; and refugees.

#### Groups implicated as African NLP technology becomes more pervasive

As technology becomes more pervasive, we must consider individuals who are yet to be born. While we cannot interview them directly, we instead invite our other stakeholder groups to consider what language technology could look like for future generations.

#### Groups likely to be overlooked by the authors

Given that the researchers of this work are African, many non-African stakeholders might not normally be considered. These include non-African expatriates working on the African continent, non-African businesses interested in selling products to African customers, and non-African governments with vested interests in Africa.

Last, all the researchers of this work are Anglophone, and so Francophone and Lusophone African stakeholders from the relevant groups need to be explicitly included.

### Personas

For each type of stakeholder within a stakeholder group, we write up a persona. Each persona is developed by analyzing interview transcripts of particular stakeholder groups. We acknowledge the diversity of the people captured by the personas and so personas themselves do not speak to a particular individual. We occasionally share fictionalized accounts from stakeholders via their personas. For each persona, we discuss the work they do; their motivation for being involved in African languages or African language technology; their training, and how their work intersects or could intersect with language technology; any challenges they face; and unique perspectives they bring to the African NLP context. Despite performing a full stakeholder identification in the previous section, the density of valuable insights we have retrieved from these interviews means we report only on the insights from the “groups involved in development of African NLP technologies.” Future work will report on insights from other stakeholder groups.

#### The content creator

The content creators support multimedia production of content. This not only covers text, but can include audio and video content as well. Depending on their professional domain, their content covers topics such as technology, health, politics, economics, digital rights and local innovation, fictional accounts, poetry, and screen plays, among other crosscutting domains. Content creators come from a variety of training backgrounds.

Content creators create with the expectation that the content is going to be consumed by audiences. They play an active role in audience selection by deciding what platforms, formats, and languages their content is made available in.

One motivation for them to create content in their Indigenous language is as a means for language acquisition for themselves and exploring a cultural heritage they feel they have lost due to colonial histories. To them, language acquisition of their Indigenous language should be better supported, and technology is a means to do this, as it has been for many other non-African languages. The process is at times stressful due to lack of available resources, and they wish for their children to not have to experience this.

Another motivation to create content in an African language is as a means of preserving the language, by continuing to revitalize its usage. Writing in an African language is a means of cultural preservation, where content creators see African language as more than a means of communication, but rather as an encoding of culture. The loss of language symbolizes the loss of the African child.

Their work is consumed by local populations, but also by members of the African diaspora, who use it as a means to connect with their communities and learn about their heritage.

The content creator sees opportunity in better tooling to support African languages, as these would serve to make their workflows more efficient and open up wider audiences for their work. They are frustrated by the inability of tools (such as spell checkers, autocompletion, and digital writing software) to adequately support their language’s diacritics and scripts or even to simply identify their own name without error.

The content creator is frustrated by the lack of digital dictionaries, thesauruses, and information about the historical and current usage of the language they are working in. They would like to know the correct pronunciation of words in the languages (and more specifically the dialects) they are writing in so that they can sing or recite them correctly, with respect for the cultural contexts.

The content creator is hindered by the lack of modern terminology for their language. This has been used against them. For example: there is no Sesotho word for feminism, and some adversaries question the legitimacy of feminism as an African concept. The content creator hearkens back to Steve Biko’s words, “The most potent weapon in the hands of the oppressor is the mind of the oppressed,” whereby the lack of support of marginalized African languages is weaponized, often by members of more historically dominant cultures, against speakers of the languages as signs of a lack of a culture’s progress.

Some content creators working for international news and media organizations that are actively growing their audiences in Africa highlight language choice as primarily being driven by populations online that are able to read and write in a particular language. This brings to light that the online population that speaks and understands a language is rarely directly correlated with the entire population of native speakers of the language, but likely a subset of a subset of it, due to factors such as literacy, access to electricity, access to technology, and finally, access to the internet that allow them to qualify as an “online” population. This may result in languages spoken by more affluent communities being better represented on digital platforms.

#### The language practitioner

The language practitioner in this work is used as a broad term for individuals who have worked in some capacity with languages, particularly in the context of developing language technology, to annotate, translate, and transcribe text or evaluate language technology systems. The language practitioner is bilingual or multilingual, which is precisely the characteristic that makes them ideal for such roles. They are proficient in one or several African languages, as well as in a Western language, typically a Western language that is a national language of their country.

(1)The linguist: the linguist has studied languages at a tertiary level and they have done substantial work focused on the African languages they speak and care about. They have often studied a second or third Western language as part of their linguistics studies. The professionally trained linguists were not aware of the opportunity to study languages at a tertiary level when much younger and were rather encouraged to aspire to undertake more traditional science, technology, engineering, and mathematics (STEM) careers viewed to be prestigious. Language careers were not as visible as STEM careers, and many language practitioners were motivated by encountering the work of other language practitioners. Among those that had the opportunity to work with both an African language and a European language, they note the stark difference in terms of availability of language tools, which subsequently affects their efficiency. Translation and transcription duties, for example, have to be done from scratch in the context of an African language, whereas for English or French, there are many tooling options, both free and paid options, which would give an initial pass of the work before requiring a human to go over and make corrections where necessary. Their awareness of this stark difference in tooling is a key contributing factor to the attitude among African language practitioners that AI tools are unlikely to replace them any time soon, as the most basic of digital tools do not even exist. One major misconception held by the general public about the linguist’s career is that anyone proficient in multiple languages can do it. Just because someone is bilingual or multilingual does not mean they will be able to translate or interpret a piece of text correctly. With the growing interest in language technologies, linguists wish the academic curriculum had introduced some computational linguistics concepts and made them aware of further possibilities at the intersection of language and technology and how their training as linguists can be useful to technologists.
(2)Annotators, transcribers, and translators: while the linguist can find career opportunities in annotating, transcribing, and translating texts, we found that, particularly in the context of language technology, many Africans, being bilingual or multilingual, find themselves also working to annotate, transcribe, and translate texts as well as evaluating language systems. This is largely due to lack of funding for professionally trained linguists to perform these activities. A language technologist will often find themselves unable to move forward with their research until a dataset is properly annotated for a particular task. Unless they have funding to hire an expert to annotate, they are likely to set out to do it themselves, since many choose to work on languages that they themselves speak, or they may reach out to their networks and communities to identify willing volunteers. More often than not, these volunteers will not be professionally trained.
It is therefore characteristic of stakeholders in the AfricaNLP ecosystem to have participated, in some capacity, as an annotator, transcriber, or translator of text despite not being professionally trained in the particular language.
(3)Evaluators: evaluators assess the correctness of a piece of language technology. They are sometimes professionally trained as a linguist, or are the language technologist themselves, ideally speaking the language in focus. The evaluator evaluates the model manually or using custom tools usually made especially for the task. Once again, we find that this role is not carried out by a distinct segment of individuals, but by a variety of people, provided they have knowledge of the language in question. (We note that in the non-African context, where work is done on African languages without the involvement of native speakers, the evaluators rarely understand the language in question and resort to existing automatic and often unsuitable evaluation metrics.^1^)

Regardless of their background, we find that a major motivation of the language practitioner is the loss of African languages. There is a sense of urgency that each African must do what they can right now to prevent this.

#### The content curator

The content curator decides what content is included into a collection, whether that collection be a dataset or a media publication. Such a content curator is motivated by the desire to appropriately preserve and represent the culture behind the language.

Their training is often in a social science discipline (in the case of media publications or museum curation) or in a computer science discipline (in the case of datasets). This contrast in expertise points to a disconnect: those trained in the social sciences might understand the impact of their curation as it represents a particular community. Conversely, those trained in a computer science or engineering background might understand the formats needed to be useful in machine learning, but are not trained to identify how the eventual corpus represents a community. While the content curator should speak the language, globally, given the move to multilingualism in NLP, it occurs quite often that the curators of the dataset do not speak the languages they protect. Conversely, initiatives like Masakhane (Masakhane Research Foundation) or SADiLaR (the South African Centre for Digital Language Resources) ensure that content is curated by individuals who do speak the languages.

The content curator would benefit from access to language tooling such as optical character recognition (OCR) tools to digitize printed works, transcription tools to ease the burden of manual transcription, and appropriate content storage that ensures that the data sovereignty of the people is respected. The content curator rarely has access to appropriate legal support when it comes to understanding and respecting copyright. Often digital text and speech are owned by large publishing corporations (e.g., BBC) and the licenses do not permit usage. The content curator is then left to negotiate with large corporations on what purposes they wish to use the data for.

Content curators who have expertise in social sciences spend significant effort in understanding how the data they collect represent the community who speaks the language and might benefit or harm the population who speaks the language. They acknowledge the power asymmetries that led to much of African history being written in English by non-Indigenous populations, whereas limited perspectives have been provided by the Indigenous African populations. Such content curators have sustainable relationships with populations who speak the language, which are built upon trust and respect. They share values with the community in ensuring that the culture and history of Indigenous African people are not lost and are instead enabled to thrive. The Indigenous sovereignty of a people who speak a particular language is of great importance to them, i.e., the people who speak the language should have the power to decide exactly what type of content represents them, their language, and their culture, as well as what happens to that content (i.e., what sort of NLP tools should be built, and by whom, and when content should be deleted, updated, or moved).

The content curator highlights that there is little work on best practices of how to store and capture text and speech data of Indigenous and marginalized populations. They note that many countries do not yet have adequate data governance laws and infrastructure in place, leading to concerns about the security and privacy of data they collect and the potential harms a data breach might have on a marginalized population.

Indigenous content curators recounted the dangers of non-Indigenous content curators who do not work closely with the community nor understand the true cultural value of the content they are curating. Such content curators (e.g., often large corporations, such as Facebook, or international researchers) extract content from the community in a way that is exploitative to those people who speak the languages. Indigenous content curators call for regulation to enforce that content curators receive training in participatory science and cultural training and be required to host collaborative workshops with a given population of speakers to understand what content they want to represent and any benefits and potential harms to a given community before proceeding to gather content.

#### The language technologist

The language technologist often, but not always, speaks the African language they choose to work on. They are often trained in machine learning, computational linguistics, or software engineering, either formally or informally, and are involved in building language technologies, based on existing datasets. Language technologists often act as content curators, since many datasets for African languages do not exist; they do so out of necessity.

The language technologists who are native speakers are motivated to work in language technologies because of the poor digital support for their language. They feel that support from technology is necessary for cultural preservation, but also to allow for continued usage and evolution of those languages.

Due to the lack of data, the language technologist is often involved in training and working with annotators for the particular annotation tasks they are interested in.

Language technologists note that their solutions often perform better than tools from large multinational companies, simply because they, the technologists, can speak the language that they are building models for and are therefore better able to curate datasets and evaluate models.

For language technologists to train better models, they actively seek collaboration with linguists who deeply grasp the language. They highlight how the tools that have been built for English were directly or indirectly informed by linguists; even if linguistic contributions were often overlooked, and to achieve similar performance in African languages, they believe linguistic knowledge will be key.

The work of the language technologist could be better supported by funding for dataset creation, since annotation is a time-consuming and expensive task, and volunteer-based annotation does not reach adequate scales. Language technologists remark that the willingness of African governments to provide support for African languages could change the future of African language technology. Language technologists note that policies around language use, such as South Africa’s official support of 11 languages and creation of a digital language resource hub, would be beneficial to other African countries and languages. The language technologists note how Africa could benefit from resources created by governments, as has happened with the EU, where all official documents are translated into European languages and official language repositories are supported (https://www.clarin.eu).

#### The language innovator

The language innovator is an entrepreneur in the African language technology space, building either for commercial or for non-profit usage. The language innovator identifies as African or as African diaspora, sometimes bringing entrepreneurial experience, training, and funding from Western society or from academia. They may also have a background in software development. The language innovator is often motivated by the difficulty of working on solutions for their language in comparison to Western languages.

Many language innovators’ focus is on three subfields:

• Applications to facilitate cultural representation, education, and celebration, such as catalogs of names (e.g., YorubaName; https://www.yorubaname.com/);
• Fundamental African language technology, such as dictionaries (e.g., Xitsonga Dictionary; https://www.xitsonga.org/dictionary), pronunciation guides, and keyboards (e.g., Bhala; https://www.bha.la/, which provides digital keyboards for the 11 South African languages);
• Speech technologies, since many African languages are very widely spoken, while being rarely written;
• Conversational NLP driven largely by corporations’ desire to build tools that allow them to better support their customers (e.g., FCB.ai, https://www.fcb.ai/; MomConnect, https://www.praekelt.org/momconnect; and MerQ, https://merq.ai/).

They are driven by a passion for their language and culture and have made significant contributions to open-source technologies in the language space. They have stumbled upon a market opportunity, particularly in the instance where they come from a software development background, and are motivated by the economic potential of building a solution. The innovator may start out focusing on one task for their product and then realize the need to work on adjacent NLP tasks due to the interconnectedness of language technologies when it comes to implementation; e.g., setting out to work on conversational AI exposes the need to work on language modeling, sentiment analysis, and semantic search, since it is likely that the tools to achieve these do not yet exist for the African language they are focusing on. If the language innovator does not have an academic or research background, they seek to collaborate with researchers who can contribute to these adjacent tasks.

The language innovator struggles to access language experts, language researchers, and funding, as well as data. They note that it is hard to scale up data annotation, transcription, and translation for African languages. They therefore begin with a focus on the language that they and their immediate community speak, acting as annotator, translator, transcriber, or evaluator, as needs be, and tapping into their community to assist with these tasks. They note that this process poses a challenge with scaling to other languages, as similar input would be required, and this might necessitate the building of a similar community that is invested in the organization/product, speaks the language, and is willing to contribute in these tasks so as to see their language supported. Alternatively, the organization would need to allocate funding to outsource these activities.

The language innovator will often begin with a national or regional market focus, driven by the language they speak and the places where the language is spoken. Another challenge cited to scaling is the multilingual nature of the African continent. While the language innovator may aspire to build a pan-African company, they acknowledge that achieving this would be an enormous feat that would require some innovative models for collaboration to make a reality.

### Themes

We highlighted and grouped closely related statements and stories from transcripts of the interviews. The commonalities from each story were extracted into subthemes, and those subthemes were grouped and categorized into the following four broad themes:

• Importance of African languages to society
• Supporting African content creation
• Creating African language technologies
• Data governance for language data

When discussing the themes, we center the perspectives captured in the interviews and expand on those perspectives. The authors furthered those perspectives by incorporating additional context and references.

#### Importance of African languages to society

A common narrative is that the world should converge to a single language of communication, and so we need not invest in diverse languages in technology. This perspective fails to acknowledge the cultural identity, Indigenous knowledge, and richness encoded in language. It also fails to acknowledge the fact that the choice of language is tied to historical oppression around the world, and by continuing to not support African languages, we continue with the artifacts of oppression. Throughout the conducted interviews, the value of African language has been emphasized.

##### The fight for the African identity

The theme of identity and the fear of its loss comes up across several of the stakeholder groups. There is a recognition that the language of a population is an inherent identifying and unifying factor, given it is the basis of communication.

Kenyan author Nanjala Nyabola states, “Language is not just words. It’s a constructed self, an orientation towards the world, an attempt to make sense of external phenomena. Language is the hands with which our minds reach into the world and try to make sense of it.”^5^ This idea of language as fundamental encoding of cultural identity is shared across Africa.

In the traditional African context, one’s mother tongue is learned in the home setting and used with family and within the linguistically homogeneous group of one’s tribe. With the increasing urbanization of African societies, the mother tongue is losing its place. The growth of African cities is unparalleled worldwide. While about 40% of the population are already living in cities today, it is expected that the 50% mark will have been crossed by 2030.^6^ Intertribal families are much more common now, and it is increasingly likely that younger generations grow up away from the hometowns and villages that their people are indigenous to and where the mother tongue is widely used for interaction.

While the emphasis shifts to enabling Africans to learn languages that are more likely to enable participation in heterogeneous African society, a somewhat latent but very real effect of this is a loss of continuity of culture, identity, and belonging.

Many Africans have adopted external cultures and ideals that are passed on to them by the custodians of the dominant Western languages that are now spoken in the African continent. There is also an acceleration of language change courtesy of urbanization and the heterogeneity of urban settings. Such language shifts due to urbanization have been studied in sociolinguistics.^7^^,^^8^ In addition, new linguistic phenomena, known as the “urban vernaculars” and “youth languages,” are emerging.^9^

The language practitioner highlights being driven by a fear of the loss of African languages and wishes they had been exposed to career opportunities in African languages much earlier in their formative education. They understand how language can encode philosophy. The linguist expresses an interest in continuing to study how languages are changing over time and particularly how they are being used on social platforms.

The content creator, by writing in their local language, creates online spaces where members of these linguistically homogeneous groups can be brought together and to some extent feel that sense of community and belonging that physical spaces may no longer provide. In addition, the content creator writes in their language to explore their heritage further, heritage that has slowly been overrun by the spread and imposition of external and popular culture.

The language technologist realizes that they have the skill to design and develop technology that can mean better support of the languages we fear losing, so they do. Even this is an act of activism, as digital spaces are inherently not designed for them; with spell checkers constantly highlighting text in our languages, autocorrect changing our names to whatever word in a Western language is statistically closest, and voice assistants requiring a squashing of our accent diversity to be understood.

Each individual is using their skills the best way they know how in the fight for the African identity, that Africans may know themselves, their people, and their origin and be able to show up as we are on digital platforms.

##### Language is key to societal participation

Language is a tool that enables interaction: interaction as natural as a child telling their parent a story, a friend asking for help, or a stranger giving directions. Language as a tool also enables participation: participation in class to reinforce learning, political participation to determine the governing of one’s country, and economic participation to earn a livelihood and support one’s family. Language is a general tool, usable within many settings. Yet participation in digital spaces, which are increasingly becoming embedded in our physical everyday spaces, has been designed such that some languages are a superior tool over others.

The language innovator saw business opportunities in the fact that companies based on the African continent with large numbers of users would like to automate functions such as customer support. The opportunity lies in the fact that existing language tools do not adequately support African languages.

We observed opportunities for enabling greater participation in African societies through better language support. The language practitioner describes their role in interpretation at public participation sessions for parliament. The content creator told a story of standing in an ATM queue. The individual at the front of the queue was getting frustrated. They wanted to withdraw money but the ATM instructions were in non-plain English. This is such a simple but explanatory example of how a lack of language support can form an economic barrier to many people.

In our multilingual societies, it is an unreasonable requirement of government to provide services such as these, which can mean actual and impactful citizen participation. Unfortunately, the scenario the language practitioner described is the exception rather than the norm. It is an expensive and labor-intensive process to achieve this level of participation, but Africans must continuously work toward it. It is an unreasonable expectation for citizens of African countries to be proficient in a major Western language for political participation.

#### Supporting African content creation

According to Nekoto et al.,^1^ to facilitate the creation of African language technologies, content in African languages needs to be created. Unfortunately, there exist many barriers in the development of language technology that are often ignored.

##### Terminology creation

Historically, speaking one’s native language was a punishable offense.^10^ It is thus not surprising that language development was halted for many African languages. Wa Thiong’o argued that Africans should enrich African languages by making them relevant for the world, and an important means of doing that is through terminology creation. Unfortunately, the acceptance of terminology in some regions is a political bureaucratic process. There are official language boards staffed by individuals who decide what words to include in the official glossaries. These official boards, according to the interviews, sometimes act as gatekeepers to the language and slow progress, which frustrates younger linguists.

This is particularly important in education, where few African languages have invested in the creation of important scientific terms. Jantjies and Joy demonstrated that students performed better when taught mathematics in their home language.^11^ The use of African languages as a means to discuss important scientific and technological progress is of utmost importance. The “Decolonise Science” project (https://www.nature.com/articles/d41586-021-02218-x), funded by the Lacuna Fund, is a practical example of creating relevant African terminology for African-centered science.

##### Basic language support for technologies

Creating digital content in African languages is frustrating due to a lack of basic tooling such as dictionaries, spell checkers, and keyboards. For languages that have diacritics, identifying and created an appropriate keyboard to use would greatly ease the process of content creation.

##### Practical implementation of language policies

Most countries in Africa have more than one national language. South Africa, for example, has 11 national languages and is mandated to have all government documents and communication translated into each of them so as to be made accessible to citizens in their preferred language of communication. The existence of such language policies is common across the world, and particularly on the African continent, where many languages are spoken. Having the policies exist, however, rarely means that they are being adequately implemented.

In the case of South Africa, parliament has a language department composed of several units whose objective is to ensure that government communication is made available in each language, as required. The translation unit largely deals with translating the Hansard, the parliamentary proceedings, as well as any written ads or communication that needs to go out to the public. The interpreting unit helps with communication as a middleman, particularly in situations where, in public hearings, a participant chooses to express themselves in a language other than English. Finally, the reporting unit transcribes everything that happens in any of the other national languages into English.

Each of these units is staffed with individuals who have undergone proper professional training in addition to training particular to the house style of parliament. All institutions have a house style that a language practitioner needs to master before they can be fully productive on their own. It is a misconception that, just because someone is multilingual, they will be able to translate or interpret a piece of text. In the case of the South African parliament, a new practitioner has to be evaluated by a senior, who acts as an editor for a period of time, until it is ascertained that they can work independently.

While these units exist, and the process to avail the needed communication is clear, the language department is often not fully staffed. This is due to a combination of factors. It is expected, but expensive, to have at least five people in each unit for each of the 11 languages. In addition, trained language practitioners are difficult to find, particularly in instances where the language has few native speakers; e.g., isiZulu is spoken by almost a quarter of the population (23%), while isiNdebele is spoken by 2% of the population.

This state of affairs presents a basis for collaboration. Government departments responsible for language policy implementation can benefit from the development of language technologies to make their processes more efficient and their labor requirements lighter. Meanwhile, language technologists can benefit from better implementation of language policies, as this would avail more data to use in their work.

##### The market for language skills in Africa

The implementation of Western languages as national languages in African countries was touted as a means to unite geographies and the diverse people that occupied various nations. The effect of these actions has been far reaching, with Western languages being used as the default for education, to conduct business, and in many other formal settings. Learning a Western language is now viewed as a necessary step to advance one’s life and career prospects, and increasingly, younger populations in Africa, particularly those born and raised in urban settings, do not learn their mother tongue.^12^ As demonstrated above, in “practical implementation of language policies,” we learn that at a macro level as well, the implementation of language policies to encourage the use of local languages for government communication and in education is lacking. Language practitioners trained in African languages, therefore, have fewer opportunities for work compared with their counterparts in other parts of the world.

This is not to say that the opportunities do not exist entirely. In South Africa, there are publishing houses who work with writers as well as translators to have books written and published in English translated to other languages. There are non-governmental organizations (NGOs) focused on children’s education who work to translate books and other educational resources. NGOs focused on issues such as gender-based violence (GBV) and HIV also work to make available resources that can be used to create awareness of these issues. Within the private sector, there are opportunities for language practitioners to translate radio and television advertisements into language that is relatable to target audiences and in the transcription of movies.

#### Creating African language technologies

##### A focus on cross-disciplinary technology careers

With a rise in interest in technology careers, and particularly the automated tools that software developers and academic researchers are building, has come a subsequent rise in the understanding of the potential risks these tools pose to society if they are developed in isolation, by non-diverse teams, and then deployed to real-world contexts.

From the personas discussed in the previous section, we have prime examples of how individuals working in disciplines considered adjacent to language technology are instrumental in the development of language tools and how these collaborations can be symbiotic.

Language practitioners already develop dictionaries and thesauruses of languages as part of their professional training. Many, however, do not have the technical know-how to make these available online and machine readable, hence, accessible for the development of language technologies, and would therefore benefit from collaboration with technologists to make this possible. On the flip side, language technologists require access to more data for the development of better-performing language tools, data that may be proprietary to content creators and language practitioners. Language technologists would also greatly benefit from the involvement of these disciplines in the evaluation cycle of tools developed, as creators and practitioners are among some of the primary customers for such tools.

Beyond general tooling for spell checking, translation, and transcription, closer collaboration with creators in the development cycle could lead to more advanced tools. Collaboration with content curators in the development of NLP tools for content moderation can lead to safer online interactions, and more broadly, collaboration with experts from different disciplines, such as anthropology and linguistics, can aid in culturally and contextually relevant output from automated language tools.

##### The importance of linguistic collaboration

The state of the art in language technology currently requires large amounts of data. Studies increasingly show that having more data leads to higher performance gains than the introduction of linguistic knowledge into the machine learning techniques popularly used. However, African languages do not have the “luxury” of copious data. Their underrepresentation further means that basic tools such as dictionaries and phonetic mappings, which would aid some machine learning techniques, do not exist.

While both technologists and linguists have an intuitive sense that they can be of use to each other, there is room to further explore what resources can be created at the intersection of the two disciplines that would be of use in building language technology, particularly using techniques that can leverage knowledge of linguistic elements of a language in lieu of access to large amounts of data. There are also linguists who have a desire to venture into language processing; however, the formal training in Africa is such that the curriculum does not expose them to the possibilities available to them in their undergraduate courses. The development of language technology in Africa would benefit from the introduction of computational linguistics concepts to linguists in their formal training.

In addition, language practitioners are an ideal first client for many of the tools currently being developed by NLP researchers, largely for research purposes. Their involvement in the development of these tools as evaluators of their performance in a real-world setting would help the researchers iterate on the tools for the development of marketable products.

##### Human augmentation

AI tools as portrayed in pop culture are thought to have the possibility to one day become more intelligent than humans. Language technologists who work to build these tools have varied opinions as to the feasibility of this. It should be noted that portrayals of AI in pop culture often refer to artificial general intelligence (AGI), which would be a machine capable of understanding the world and with a capacity to learn similar to that of a human. The AI tools referred to in this work, language tools, are applications of AI technology in a narrow domain. It is these applications of AI that are currently widely applied in business to optimize various narrow functions in sectors such as finance and communication. This optimization has in some cases rendered human labor redundant and fueled the perception of AI as a technology that will lead to widespread job loss and a redefinition of work in and of itself, the future of work.

The language practitioner expressed confidence in their job security, demonstrating that the lack of focus on language tools for African languages in particular means that a lot of the tasks they carry out professionally need to be done quite manually, the few tools that do exist perform relatively poorly, and it will take a significant amount of time before they feel at risk of being replaced. The language technologist realizes that the current development of AI tools is largely driven by big tech, private organizations that are primarily profit driven. This, therefore, means that the ways in which they have chosen to design AI is with an emphasis on efficiency first and dealing with the effects on human labor and society only after. There is room to do things differently. It is possible for AI practitioners to design AI tools while being conscious of the contexts within which they will be deployed. In the context of language, the authors recommend language technologists to work with the professionals who are adjacent to language and to collaborate with them to build tools that aid the professionals’ work and augment their ability so that human intelligence and participation can remain central to the deployment of the AI tools.

The future of work in Africa needs AI tools that are designed to help individuals unlock greater potential. For the language practitioner and content creators, that means access to the fundamentals, spell checkers, dictionaries, and keyboards. It means reaching out to these adjacent communities and asking them what else in their process could be made more efficient with AI tools and ensuring that they remain central to the design and development process.

#### Data governance for language data

##### Data collection

Dataset collection should be intentional, rather than attempting to scrape anything available. Data collection can also be reinterpreted, in the African NLP context, as data creation, since often the data do not exist yet in digital form. Not only should one be intentional about what is collected, but also who the speaker or author is and how that data collection benefits the communities for which the data are collected. In addition, data collection should respect the privacy of the authors (especially in the case of speech data).

##### Dataset curation

Dataset curators typically come from a computer science or linguistics background. The problem is that computer science and linguistics training does not traditionally include skills required for modern dataset curation, especially when those datasets are to be used by machine learning models. In fact, little research exists on how to curate datasets so that they minimize harm, are representative of the communities, and abide by ethical data collection practices.

##### The duality of open datasets

The open data movement aims to enable better scientific experimentation but also democratize the data for greater use by organizations.

In the African context, there exists a duality when opening up a dataset. While the accessibility of the dataset will spur further research by both Africans and non-Africans, it also means that commercial value can be sought by both Africans and non-Africans. In other words, a non-African company can profit from African data and often exploit the Africans who opened up the data.

Nekoto et al.^1^ describe how low resourcedness is not only about data, but is rather low resourcedness at a societal scale, in terms of compute, funding, and support. Simply because the data are open does not mean that everyone has equal opportunity to benefit from them. An organization that has financial support for their language technology operations (such as Facebook), is more likely to be able to benefit and profit from African language data than Africans. Power asymmetries cannot be ignored, even when talking about open data.^13^

### Recommendations

We would like to put forward the following recommendations for stakeholders working in the African language ecosystem. This includes all stakeholder groups identified in this work through the Diverse Voices methodology^4^ and policy makers:

• Language acquisition of Indigenous African languages, primarily by Africans, should be better supported, and technology is a means to do this, as has been the case for many other non-African languages.
• Basic tooling to support content creation on digital platforms, such as digital dictionaries, thesauruses, keyboards supporting diacritics where relevant, and spell checkers that recognize African names and places without error, should be prioritized.
• Language tools and processes for content moderation and to catch and control the spread of misinformation online in Indigenous African languages should be developed and actively used.
• Language careers and the professional opportunities available, particularly as pertains to Indigenous African languages, should be made more visible to students earlier in their education so as to generate greater interest in these fields in tertiary education.
• AI language tools that augment human activities as opposed to tools seeking to replace them should be the intentional design choice, especially given the current dearth of tooling and data for African languages.
• Computational linguistics components should be introduced into the educational curricula of disciplines adjacent to and working with language, e.g., linguistics and journalism, with an emphasis on the role they can play in the development of ethical and inclusive AI so as to encourage a pipeline of cross-discipline stakeholders working to build language technology.
• Professional training opportunities to enable multilingual individuals to venture into language careers should be increased.
• The study of contemporary use of language in Africa should be emphasized, given increasing urbanization and the multicultural nature of the continent.
• Funding for dataset creation and annotation, both of which can be time-consuming and expensive tasks, should be increased.
• African language policies, particularly those pertaining to education and provision of government services, should be better implemented with the aid of emerging language tools and technologies.
• Digital licensing and funding should be made suitable to support legal cases against non-African corporations who use open African data.
• An “ethical data curation toolkit,” which is informed by information scientists, data privacy experts, and machine learning bias experts, would empower dataset curators with the knowledge and skills to perform informed data curation. The toolkit should be accompanied by a workshop in which practical training and discussions can take place.

### Limitations of the study

This study focuses on interviews with limited participants, which may not be representative of the whole community. Future work would include a larger quantitative study of the community.

## Experimental procedures

### Resource availability

#### Lead contact

Further information and requests for resources should be directed to and will be fulfilled by the lead contact, Kathleen Siminyu (kathleensiminyu@@gmail.com).

#### Materials availability

The full list of stakeholders identified within the AfricaNLP ecosystem, as well as the questionnaire used in this work are included in the supplemental experimental procedures.

## Data Availability

This paper does not report original code. The qualitative data collected from interviewing stakeholders is reported in the paper.

## Supplemental information

Document S1. Supplemental experimental procedures

Document S2. Article plus supplemental information

## References

[1] Nekoto W., Marivate V., Matsila T., Fasubaa T., Kolawole T., Fagbohungbe T., Akinola S.O., Muhammad S.H., Kabongo S., Osei S. (2020). Participatory research for low-resourced machine translation: A case study in african languages. arXiv.

[2] Siminyu K., Martinus L., Marivate V. (2020).

[3] Abbott J., Kreutzer J., Elsahar H., Siminyu K., Marivate V., Subramani N., Duvenhage B. https://sites.google.com/view/africanlp-workshop.

[4] Magassa L., Young M., Friedman B. (2017). Technical report.

[5] Nyabola N. (2018).

[6] Un-Habitat (2012).

[7] Söylemez Ü. (2004). Urbanization and language shift in turkey: the change processes at work in the transition from rural to urban settings. Int. J. Sociol. Lang..

[8] Tandefelt M. (1994). The Sociolinguistics of Urbanization.

[9] Beck R.M. (2010). Urban languages in africa. Afr. Spectr..

[10] Wa Thiong’o N. (1992).

[11] Jantjies M., Joy M. (2016). Lessons learnt from teachers’ perspectives on mobile learning in south africa with cultural and linguistic constraints. S. Afr. J. Educ..

[12] Kaiper A. (2018). if you don’t have english, you’re just as good as a dead person”: A narrative of adult english language literacy within post-apartheid south africa. Int. Rev. Educ..

[13] Abebe R., Aruleba K., Birhane A., Kingsley S., Obaido G., Remy S.L., Sadagopan S. (2021). Proceedings of the 2021 ACM Conference on Fairness, Accountability, and Transparency.

