# Supplementary material for: Consultative engagement of stakeholders toward a roadmap for African language technologies
Source: Patterns (N Y). 2023 Aug 11;4(8):100820. doi: 10.1016/j.patter.2023.100820 (PMC10436040; doi:10.1016/j.patter.2023.100820)
Supplement: Document S1. Supplemental experimental procedures [file mmc1.pdf]

Patterns, Volume *4*

Supplemental information

Consultative engagement of stakeholders toward a roadmap for African language technologies

Kathleen Siminyu, Jade Abbott, Kọ́lá Túbọ̀sún, Aremu Anuoluwapo, Blessing K. Sibanda, Kofi Yeboah, David Adelani, Masabata Mokgesi-Selinga, Frederick R. Apina, Angela Thandizwe Mthembu, Arshath Ramkilowan, and Babatunde Oladimeji

Roadmap for AfricaNLP

Authors: Kathleen Siminyu and Jade Abbott

Document Objectives

Our goal is to:

- Discover and outline further research avenues, investment and impact potential of the groundwork now being funded and scaled to be applicable across Africa for African languages
  - Identify core research topics that might catalyse progress in African NLP, eg. performance metric specifically developed for African languages
  - Identify platforms and tooling that can incentivize the scaling of this work to more African languages
  - Identify partners and investors from different sectors that can collaborate both in the research and in the implementation of impact potential work
- Identify stakeholders, drivers and foci for strategies, roadmaps, and business plans and positions and opportunities in order to incorporate new developments in African language
  - Explore multi-disciplinary relationships with external stakeholders of the African NLP ecosystem; content creators(writers, musicians), translators, curators(publishers), for strategic development and use of NLP tools and context relevant data
  - Explore the economic feasibility of productization of NLP tools across various markets in Africa and recommend avenues for the support of local innovation and enterprise in the area
  - Explore opportunities for collaboration with scholars and researchers focused on the pedagogy of African languages, for greater exposure and accessibility to tools and resources for learning of african languages
  - Explore opportunities for collaboration with stakeholders that can help ensure ethical and responsible development of AI tools
- Describe the structures and relationships required to maintain and extend the Fellowship's valuable offering beyond 2021
  - Recommend avenues for support from UNESCO country offices, other relevant national and regional organisations

Methodology

The roadmap is to be compiled in a collaborative fashion.

1. Outline the major objectives of the document
2. Outline the technologies of interest within the African NLP community

3. Outline the stakeholders of interest where the technologies are concerned as well listing some questions to speak to them about
4. Open up the document for high level consultation with members of the Masakhane community and potential partners
5. Compile a list of stakeholders from the community and potential partners and schedule meetings
6. Gather input from stakeholders via an interview process
7. Distil insights and incorporate them into the roadmap
8. Determine whether further input is needed from experts and conduct a subsequent round of interviews. This would be necessary in the event that neither of us is conversant with a topic that recurs in conversation with interview participants.
9. Finalize v1 of the AfricaNLP Roadmap

Technology of Interest

Natural Language Processing describes a group of technologies which engage with how technology can process, understand, and generate natural language and speech. There are a variety of NLP tasks that have gained a fair amount of popularity, such as:

- Sentiment Analysis - understanding the emotion communicated by a piece of text
- Machine Translation - the ability for a machine to translate between languages
- Speech technologies - the ability for a machine to understand spoken word, or generate spoken word
- Named entity recognition - the ability for a machine to identify real world entities in speech
- Information retrieval - the ability for a machine to retrieve answers when queried
- Topic Modeling - the ability of a machine to classify document by different topics

Unfortunately, despite Africa having over 2000 languages, little work has been done to address NLP for African languages. This is often attributed to the lack of African language data that is available. However, systematically evaluating the cause of why this is the case, it is clear that most African languages do not have key language technologies (e.g. digital keyboards, online dictionaries, thesauruses, spell checkers, auto-completion). Without these tools, the digitization of African languages is hindered, and thus so is the process of building language technologies. ∀ (2020) refer to a virtuous cycle which emerges when the correct resources, demand and connections between stakeholders exists

Stakeholders

Our strategy for identifying stakeholder groups is adapted from the Diverse Voices methodology. We look into 4 types of stakeholders:

- Group 1: Those likely to use the technology of interest.
- Group 2: Those not likely to use the technology of interest due to factors such as structural inequality in society, disinterest, or self-described technophobia
- Group 3: Those that are likely to be overlooked, based on the groups represented by the facilitators and/or document authors
- Group 4: Those that will be implicated as the technology becomes more pervasive in the future

Group 1: Those who are likely to use the Technology of Interest

We divide Group 1 into 3 groups: Creators; Users; Indirect. This division stemmed from expanding out from those closest to the technology, to those furthest from the technology

Group 1.1: Creators

Who are they

Creators are stakeholders who interact with the technology by **creating** it, as outlined by ∀ (2020). This group includes individuals who are in the **natural language sector** who are also the most at risk to be replaced by the technology.

a. **Content Creators** produce content in or about a language, where content is any digital or non-digital representation of language. In society, these would be:
    - Journalists
    - Copywriters
    - Creative writers (song writers, poets, authors)
    - Technical writers
    - Linguists

b. **Annotators/Transcribers/Translators** annotate, transcribe or translate the content including crowd-workers, researchers, or transcription/translation professionals. They must understand the languages involved

c. **Curators** are defined as individuals involved in the content selection for a dataset requiring access to content and translations. They should understand the languages in question for quality control and encoding information.
    - Editors
    - Dataset Creators
    - Publishers
    - Data Governance Experts

d. **Language Technologists** are defined as individuals using datasets and computational linguistic techniques to produce NLP models between language pairs. Language technologists require language preprocessors, NLP toolkits, and access to compute resources.
    - NLP practitioners
    - Software engineers
    - Academic Researchers

(Including those working on non-African low-resource languages)

e. **Evaluators** are individuals who measure and analyse the performance of an NLP model, and therefore need knowledge of both source and target languages. To report on the performance on models, evaluators require quality metrics, as well as evaluation datasets. Evaluators provide feedback to the Language Technologists for improvement. Beyond performance evaluation, evaluators are also individuals who critique the work of language technologists using relevant decolonial, ethical and gender equity frameworks to ensure the resulting models are not likely to perpetuate human biases.

f. **Language Innovators(Entrepreneurs)** are individuals who are using language technologies to solve problems in various contexts. They are creating or identifying markets for these technologies and building organisations to refine, package and productize these language tech services/offerings at a cost and for profit.

What we hope to learn from them

1. Questions for ***Content Creators***
   a. What kind of content do you create? (ask to specify languages)
   b. What is your understanding of language technology?
   c. Who is your audience? Are they largely based in Africa or globally?
   d. On what platforms is your content available on?
      - Do you monetize your content?
   e. What language(s) is your content accessible in?
      - Do you think this has been a determinant of who your audience is/has been?
   f. Can you give us a brief overview of your content creation process in terms of major stages involved and the technical tools, if any, you use at various stages of your work.
      - Do you have any thoughts on how these tools can be improved to make your work more efficient?
      - Are there other tools you wish existed that would make your work more efficient?
   g. Are there tools you already use/think you could use to make your content more accessible to audiences that speak different languages?
      - What about audiences that are differently-abled?
   h. Given your understanding of language technology, what do you believe is the future of your work?
2. Questions for **Annotators/Transcribers/Translators**
   a. What is your career?
   b. What tools do you use in your line of work?
      - What is your understanding of language technology?
   c. What are your motivations for being in this career?
   d. Have you performed any annotation/translation/transcription work?
      - What sort of annotation/transcription/translation have you performed?
   e. Was this work paid or volunteer work?

        - If funded,
            - Are you comfortable sharing who funded it?
            - Are you comfortable providing a range for which you are paid for such work?
            - Do you think the rate you were paid was fair?
    f. Can you discuss your process and the technical tools (if any used) by the milestones and phases of your work?
        - Do you have any thoughts on how these tools can be improved to make your work more efficient?
        - Are there other tools you wish existed that would make your work more efficient?
    g. Given your understanding of language technology, what do you believe is the future of your work?
3. Questions for ***Content Curators***
    a. What kind of content do you curate?
    b. What language(s) is this content available in?
    c. What platform(s) is this content available on?
    d. What is your understanding of language technology?
    e. Can you give us a brief overview of your curation process in terms of major stages involved and the technical tools, if any, you use at various stages of your work.
        - Do you have any thoughts on how these tools can be improved to make your work more efficient?
        - Are there other tools you wish existed that would make your work more efficient?
    f. Are there tools you already use/think you could use to make your content more accessible to audiences that speak different languages?
        - What about audiences that are differently-abled?
    g. What support do you need?
        -
    h. Given your understanding of language technology, what do you believe is the future of your work?
4. Questions for ***Language Technologists***
    a. What are you working on?
        - What methodologies, techniques and/or tools do you use in your work? What specific tasks do you use them to perform and why?
    b. How long have you been working on it?
    c. Why are you working on it? What is the incentive for your work?
    d. What outcomes do you hope to see from it?
    e. What future research directions, related to your work, can you identify?
        - Are you keen to keep working on this in a research capacity?
        - If yes, what kind of support do you think you would require?
    g. Can you identify any direct/indirect users or beneficiaries of your work? How would they benefit? Is there economic value that can be created?

        - Are you keen to keep working on this in a bid to capture that economic value?
        - If yes, what kind of support do you think you would require?
    h. Are there any multi-disciplinary relationships/collaborations that you think could/would greatly advance the work you are involved in?
5. Questions for ***Evaluators***
    a. What language models do/have you evaluated?
        - Please describe the process of carrying out this evaluation, particularly wrt interaction with the language technologists
    b. What metrics or frameworks do you use in your work?
    c. Do you actively engage in research to develop better metrics and frameworks for evaluation?
    d. What were the challenges faced in evaluation?
    e. What support?
    f.
6. Questions for ***Language Innovators(Entrepreneurs)***
    a. What language-related venture are you working on?
        - What language technologies are you making use of?
        - What language(s) are you supporting?
    b. How long have you been working on the venture?
    c. Can you speak about who your target users are?
    d. Have you had any paying customers thus far?
        - If yes, can you describe a general profile of your customers?
    e. Do you actively engage in research to improve your services?
    f. Do you have plans to eventually scale, either in terms of the number of language services that you offer or in terms of language/geographic coverage?
    g. Have you raised any funding?
        - If no, is it something you might do in future?
    h. What challenges?
    i. What support?

Group 1.2: Users

Who are they

Users are stakeholders where language ***is relied upon*** in order to perform their job, but is not the core focus on the job: Educators; Legal; PR; Customer Service; Media; Government; Health; Commerce; Market Research; Research; the hearing or eyesight impaired.

What we hope to learn from them

Note: Prof Marwala will be a meta-interview

1. What is the nature of your work/occupation?

2. What languages do you speak?
    a. Are you able to navigate work/life/education in these language(s)?
    b. Are you able to access government/social services with knowledge of these language(s)?
        - If no, what are the barriers to access?
3. What is your understanding of language technology?
    a. Given your understanding of language technology, what do you think is the future of work in your industry, augmented by language technology?
4. What languages do you write?
5. Describe circumstances where you are unable to perform a task (read, write, listen, search, speak) in a language that would have aided your job?
6. Do you interact with any technology devices often?
    a. If yes,
        - what technologies/platforms?
        - how do you interact with them?
    b. If no,
        - why not?
7. Do you access the internet regularly?
    a. If yes,
        - what for?
        - on what devices?
        - how often?
    b. If no,
        - why not?
8. Do you know of any technology solutions beyond your reach that would/might improve your quality of life in any way?

Group 1.3: Indirect

Who are they

Indirect stakeholders are stakeholders who are impacted by the NLP technologies, but do not necessarily engage with the technology itself and instead engage with the **artifacts** generated by the technology

- African students (who can use translated resources, better search, etc)
- General African public (by legal, government, media, health providing translated or more accessible documents)
- General African public (who use social media)
- African technology users

What we hope to learn from them

1. What languages do you speak?

2. What languages do you write?
3. Describe circumstances where you are unable to perform a task (read, write, listen, search, speak) in the language you're most comfortable in
4. Do you have access to technology, and if so what? and how do you engage with that technology? What do you use it for?

Group 2: Those who are not likely to use the technology of interest due to factors such as structural inequality in society, disinterest, or self-described technophobia

Who are they

In order to identify which stakeholders are not likely to use the technology of interest, we consider a number of **accessibility dimensions**, and use that to help identify such populations.

Our identified **accessibility dimensions** are as follows

- Income (device & connectivity costs)
- Internet access (function of location & income)
- Literacy (access to education) - for text NLP
- Digital literacy
- Hearing Ability - for speech
- Eyesight ability - for text
- Language (their language would need to be supported)

We use the above dimensions to identify marginalized groups that are often left out of the technology conversation:

- African individuals below the poverty line
- Individuals relying on spoken or undocumented languages
- Those not connected to the internet (given only 26% of Sub-saharan Africa is connected via mobile as of 2019)
- The illiterate
- The elderly
- The youths
- Women, especially rural women (gender gap in digital literacy & internet connectivity)
- Mothers, especially single or working mothers
- Cultural custodians (African tribal leaders)
- The unemployed
- The incarcerated
- People with disabilities
- Illegal immigrants
- Social workers
- Those living under oppressive governments
- Refugees

- Illegal immigrants

What we hope to learn from them

1. What is the nature of your work/occupation?
2. What languages do you speak?
    a. Are you able to navigate work/life/education in these language(s)?
    b. Are you able to access government/social services with knowledge of these language(s)?
        - If no, what are the barriers to access?
    c. Describe any circumstances where you are unable to perform a task (read, write, listen, search, speak) in the language you're most comfortable in.
3. Do you interact with any technology devices often?
    a. If yes,
        - what technologies/platforms?
        - how do you interact with them?
    b. If no,
        - why not?
4. Do you access the internet regularly?
    a. If yes,
        - what for?
        - on what devices?
        - how often?
    b. If no,
        - why not?
5. Do you know of any technology solutions beyond your reach that would/might improve your quality of life in any way?

Group 3: Those that are likely to be overlooked, based on the groups represented by the facilitators and/or document authors

Who are they

- non-African society & business:
    - non-African expatriates working on the African continent (via NGO or Commerce)
    - African diaspora not working on the African continent.
    - non-African businesses interested in selling or provided customer support for their services in Africa
    - non-African governments interested in understanding African people more
- Francophone and Lusophone Africa stakeholders from each of the relevant groups

What we hope to learn from them

1. What is the nature of your work/occupation?
2. Have you ever needed to engage read/write/speak/understand an African language? If so, please describe the scenario
3. Do you know of any language technology solutions beyond your reach that would/might improve your quality of life in any way?

Group 4: Those that will be implicated as the technology becomes more pervasive, possibly decades in the future

Who are they

Children and populations decades in the future who are potentially yet to be born.

What we hope to learn

Whether the other stakeholders consider future populations in their design of these technologies and how they envision their work will affect them.

How Can You Help?

- Feedback on this document would be appreciated.
- If you can think of stakeholders that fit into either of the above 4 groups that we should speak to, please fill out this form leaving us some info of why you think their input valuable and their contact information if possible. (You can also nominate yourself)

About the Interviews

- Interviews will take at least 40 minutes, an hour at maximum, and will be on Zoom. Other platforms are also possible for the call if you have a particular preference.
- Interviews will be recorded for transcription purposes and then deleted; there is the option to not have your interview recorded. You can also choose to have only the audio recorded and not the video. If you are not comfortable with either, or one, then we will figure out a workaround, like taking notes while we speak.

References

[1] 3 reasons why most Africans aren't on the internet – and how to connect them
[2] Mobile Internet Connectivity 2020 Sub-Saharan Africa Factsheet
